# Rubber band ligation combined with clip-assisted loop preligation for removal of a difficult-to-access gastric fundus tumor

**DOI:** 10.1055/a-2971-3935

**Published:** 2026-10-12

**Authors:** Lingfeng Ruan, Ling Zhang, Zunling Chen, Yiqian Liang, Weiyi Zhou, Qingyu Zeng, Zhang Tao

**Affiliations:** 1 Department of GastroenterologyAffiliated Hospital of Southwest Medical University; Nanchong Central HospitalNanchongSichuanChina; 2 Department of GastroenterologySouthwest Medical University, Nanchong Central Hospital.LuzhouSichuanChina

A 56-year-old woman was found to have an elevated lesion in the gastric fundus during routine endoscopy. Endoscopic ultrasonography revealed a heterogeneous hypoechoic lesion that appeared to arise from the muscularis propria. No tissue was obtained during the preoperative endoscopic ultrasonography examination. A mesenchymal tumor was suspected, with a gastrointestinal stromal tumor and leiomyoma considered in the differential diagnosis. Because the conventional endoscopic resection of lesions at this location carries risks of perforation, extraluminal bleeding, incomplete resection, and tumor spillage into the peritoneal cavity, 1​ ​2​ tumor resection was performed using rubber band ligation combined with clip-assisted loop preligation ( Video 1 ). Endoscopy revealed a smooth, elevated lesion in the gastric fundus ( Fig. 1 ). The lesion was suctioned into a white cap and ligated with a rubber band ( Fig. 2 ). A loop was then prepositioned around the base of the lesion and secured with clips to achieve clip-assisted loop preligation before resection ( Fig. 3 ). The tumor was subsequently resected en bloc above the loop using a hot snare. The resection site was clearly visualized, with no evidence of bleeding or perforation ( Fig. 4 ), and additional clips were applied for closure. The resected specimen measured 1.2 cm×1.0 cm ( Fig. 5 ), and histopathological examination confirmed a spindle-cell-type gastric gastrointestinal stromal tumor. A postoperative upper gastrointestinal contrast study showed no evidence of leakage. The patient was discharged on postoperative day 4 without adverse events. Follow-up endoscopy 1 month later showed good healing of the resection site.

**Video 1** Rubber band ligation combined with clip-assisted loop preligation for the removal of a difficult-to-access gastric fundus tumor. Video URI:


**Fig. 1:**
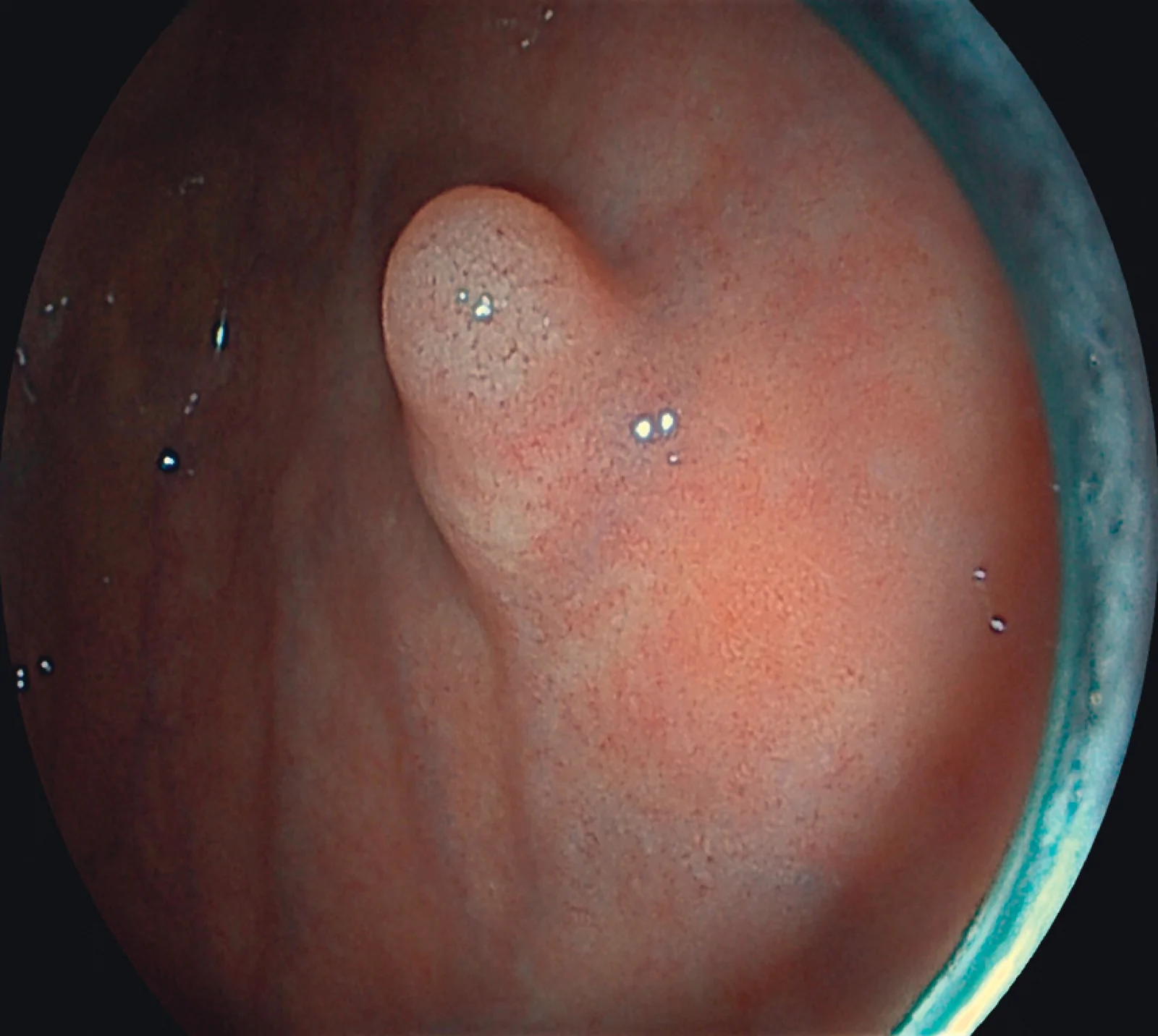
An endoscopic view showing a smooth, elevated lesion in the gastric fundus.

**Fig. 2:**
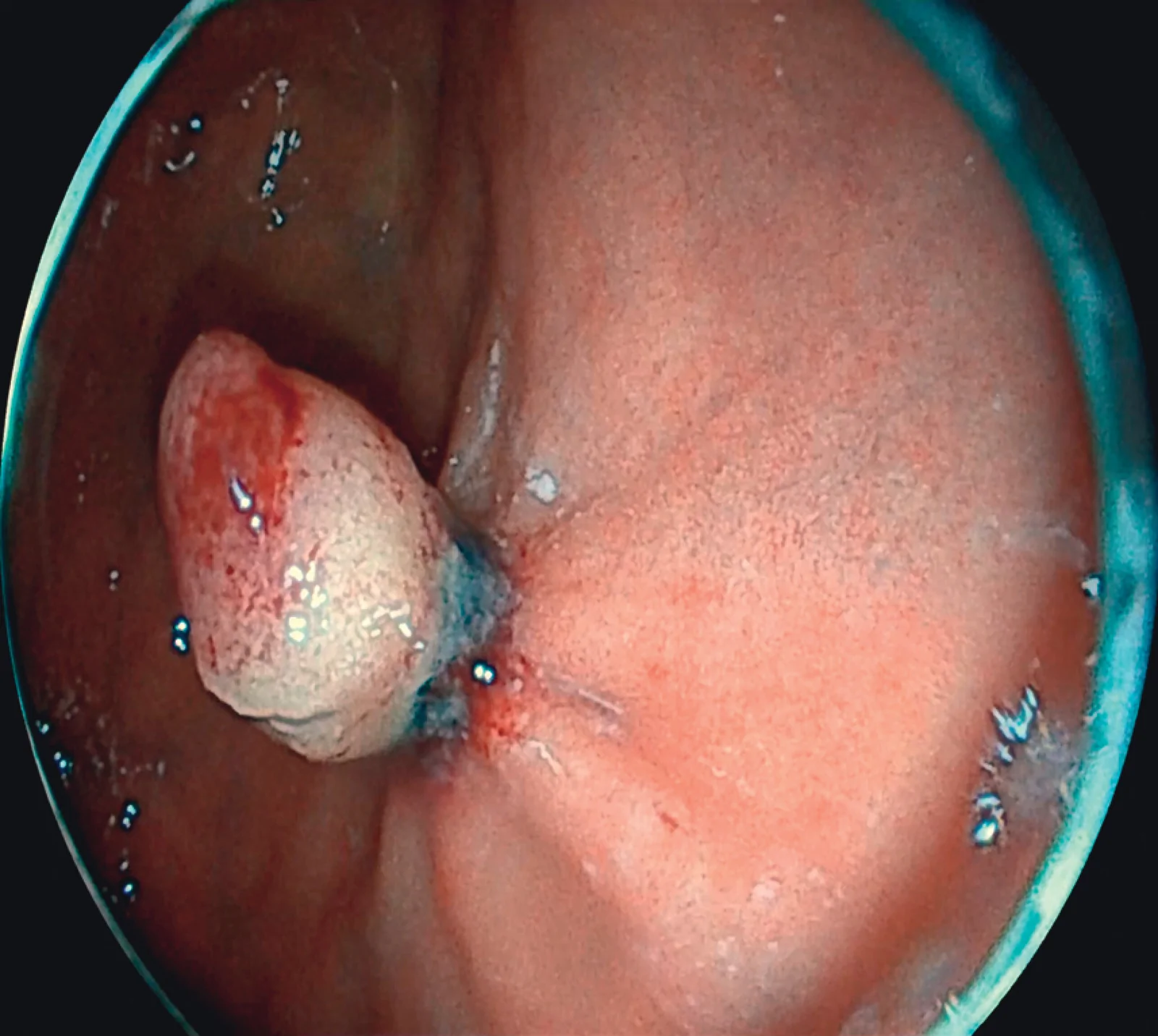
The lesion was suctioned into a white cap and ligated with a rubber band.

**Fig. 3:**
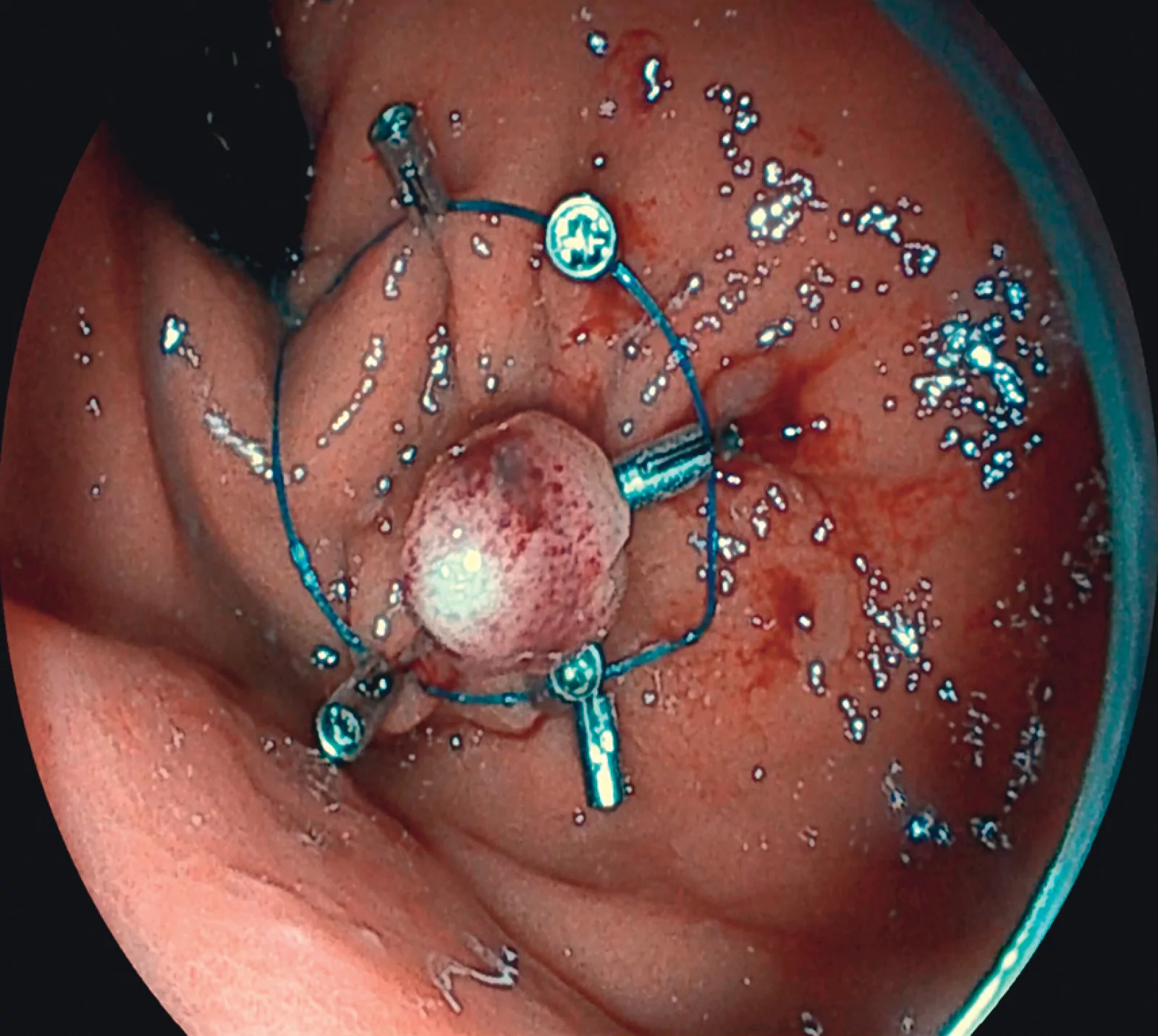
A loop was prepositioned around the base of the ligated lesion and secured with multiple clips to achieve clip-assisted loop preligation before resection.

**Fig. 4:**
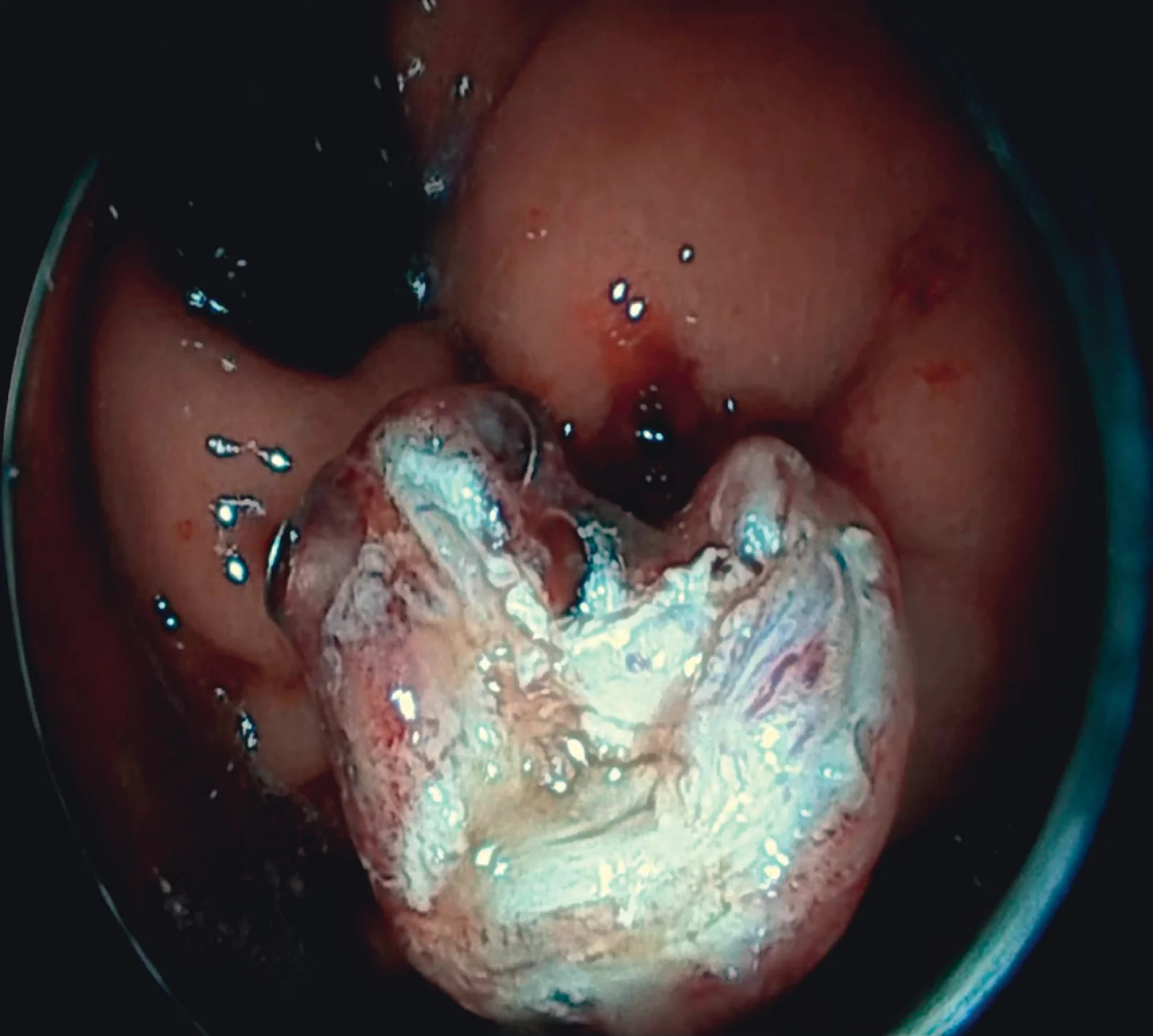
The resection site was clearly visualized after tumor removal, with no evidence of bleeding or perforation.

**Fig. 5:**
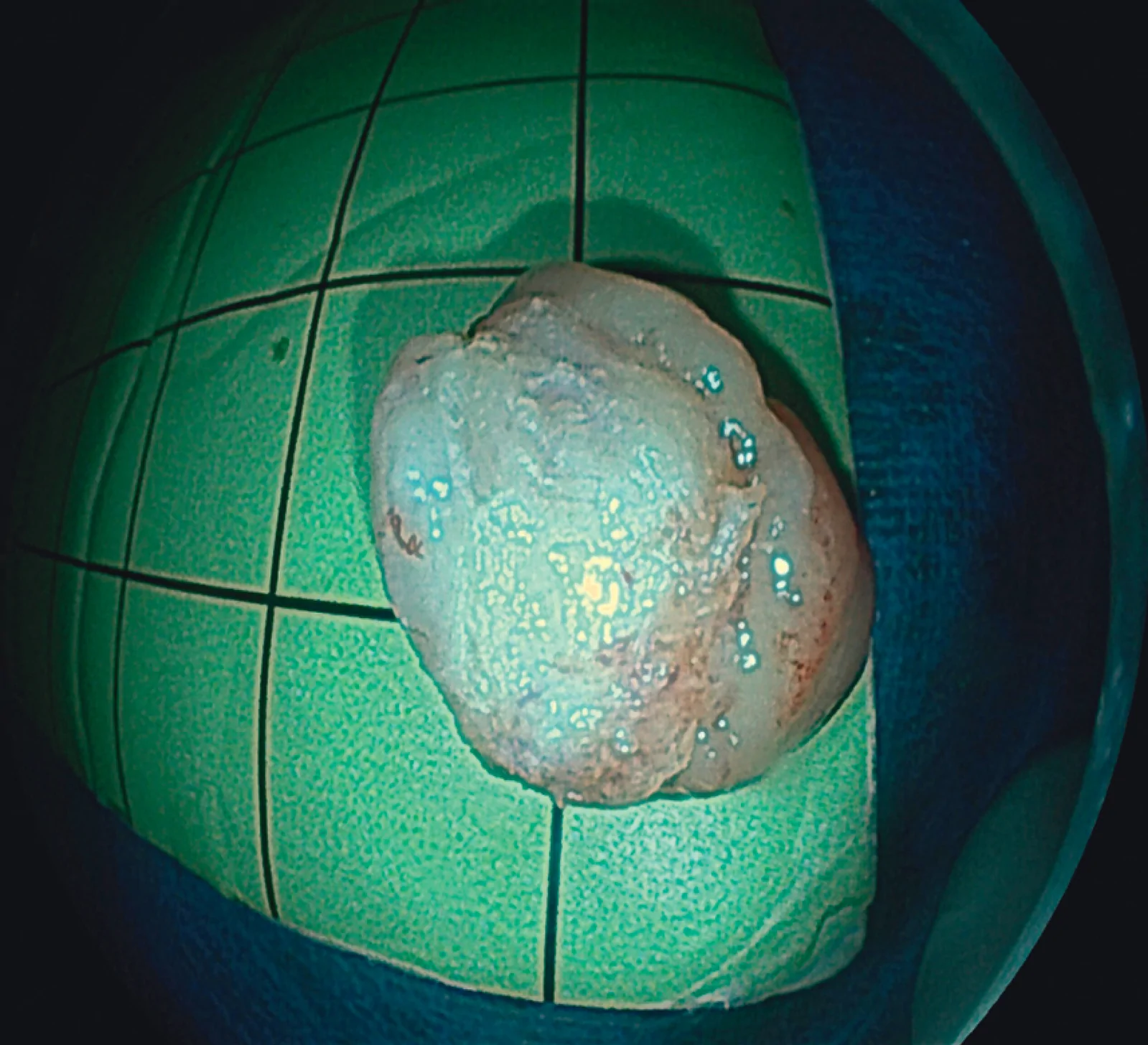
An en bloc resected specimen measuring 1.2 cm×1.0 cm.

Endoscopic treatment of tumors arising from the muscularis propria in the gastric fundus is challenging because of the retroflexed approach, thin gastric wall, and limited maneuverability. 3​ ​4​ Compared with conventional techniques, such as endoscopic submucosal dissection and excavation, 1​ ​2​ this approach may be simpler, less costly, and less time-consuming. Preligation of the tumor base may facilitate the management of the resection site by providing traction and stability, improving procedural control, and reducing procedure-related risks. These advantages suggest that this technique may be particularly useful for difficult-to-resect tumors from the muscularis propria in the gastric fundus.

Endoscopy_UCTN_Code_TTT_1AO_2AG_3AD
